# Community response to intermittent preventive treatment of malaria in infants (IPTi) in Papua New Guinea

**DOI:** 10.1186/1475-2875-9-369

**Published:** 2010-12-22

**Authors:** Christopher Pell, Lianne Straus, Suparat Phuanukoonnon, Sebeya Lupiwa, Ivo Mueller, Nicolas Senn, Peter Siba, Marjolein Gysels, Robert Pool

**Affiliations:** 1 Barcelona Centre for International Health Research (CRESIB), Hospital Clínic / IDIBAPS, Universitat de Barcelona, Spain; 2 Papua New Guinea Institute of Medical Research, Madang MP511, Papua New Guinea; 3 Swiss Tropical and Public Health Institute, Socinstr. 57, 4051 Basel, Switzerland; 4 University of Melbourne, Melbourne, Victoria 3010, Australia; 5 Centre for Global Health and Inequality, University of Amsterdam, Spui 21 1012 WX Amsterdam, The Netherlands

## Abstract

**Background:**

Building on previous acceptability research undertaken in sub-Saharan Africa this article aims to investigate the acceptability of intermittent preventive treatment of malaria in infants (IPTi) in Papua New Guinea (PNG).

**Methods:**

A questionnaire was administered to mothers whose infants participated in the randomised placebo controlled trial of IPTi. Mothers whose infants participated and who refused to participate in the trial, health workers, community reporters and opinion leaders were interviewed. Men and women from the local community also participated in focus group discussions.

**Results:**

Respondents viewed IPTi as acceptable in light of wider concern for infant health and the advantages of trial participation. Mothers reported complying with at-home administration of IPTi due to perceived benefits of IPTi and pressure from health workers. In spite of patchy knowledge, respondents also demonstrated a demand for infant vaccinations and considered non-vaccination to be neglect. There is little evidence that IPTi has negative impacts on attitudes to EPI, EPI adherence or existing malaria prevention practices.

**Conclusion:**

The degree of similarity between findings from the acceptability studies undertaken in sub-Saharan Africa and PNG allows some generalization relating to the implementation of IPTi outside of Africa: IPTi fits well with local health cultures, appears to be accepted easily and has little impact on attitudes towards EPI or malaria prevention. The study adds to the evidence indicating that IPTi could be rolled out in a range of social and cultural contexts.

## Background

Intermittent preventive treatment (IPT) of malaria involves the administration of treatment doses of an anti-malarial drug at predetermined intervals, regardless of parasitaemia or symptoms. IPT during pregnancy (IPTp) is linked to ongoing routine antenatal care and IPT for infants (IPTi) is delivered through the Expanded Programme of Immunization (EPI) [1].

Various studies in sub-Saharan Africa have shown that IPTi with sulphadoxine-pyrimethamine (SP) given at the time of routine vaccinations in the first year of life reduces the incidence of clinical malaria by between 20% and 59% [2-7], and by 30% across the six sites [8]. In addition to the clinical effectiveness of IPTi, recent social science research has also demonstrated that in several sites across sub-Saharan Africa, IPTi is socially and culturally acceptable [9-11]. This acceptability research, undertaken alongside clinical trials (in Gabon, Kenya, Mozambique and Tanzania) and implementation studies of IPTi (in Ghana, Malawi and Tanzania) with various drug regimens, also indicated that IPTi does not negatively influence attitudes to and uptake of immunization, nor is IPTi misunderstood as immunization against malaria. IPTi does not, therefore, influence other preventive measures or delay treatment seeking for malaria.

Although the acceptability research published to date provides a comprehensive insight into community responses to IPTi delivered in sub-Saharan Africa, there are no published data on the acceptability of IPTi outside of sub-Saharan Africa. Given that IPTi has the potential to reduce the malaria-related morbidity and mortality for infants in other regions with a significant level of malaria mixed endemicity (*Plasmodium falciparum *and *Plasmodium vivax*), such as South-East Asia or Oceania, it is crucial to ensure that IPTi is socially and culturally acceptable in distinct social and cultural settings outside of sub-Saharan Africa.

Building on previous acceptability research undertaken in sub-Saharan Africa and in the context of an IPTi trial in Papua New Guinea (PNG), this article aims to:

1. Describe knowledge, perceptions, experiences and responses relating to IPTi and EPI of trial participants, community members, and local health care providers

2. Identify and understand the mutual interactions between perceptions of, attitudes to and experiences with EPI and IPTi.

3. Identify and understand local barriers to the acceptance of and long-term adherence to IPTi.

4. Identify wider socio-cultural, national and regional factors that affect, or may affect, the implementation or acceptability of IPTi.

### Setting

The acceptability research undertaken in PNG was carried out alongside a randomized placebo-controlled clinical trial of IPTi, which was run by the Papua New Guinea Institute of Medical Research (IMR) and carried out under the auspices of the IPTi Consortium with the financial support of the Bill and Melinda Gates Foundation. The trial tested the following drug regimens: single dose of SP associated to three days of either amodiaquine or artesunate given four times at three, six, nine and 12 months following the PNG EPI schedule. Each dose of the three-dose regimen was given on consecutive days: trial staff gave the first dose; the second and third doses were given to mothers to administer to their infants the following days. The study drugs were administered in collaboration with local health facilities responsible for operating Mother and Child Health (MCH) clinics, which, due to the scattered nature of settlements in PNG, deliver EPI through monthly village outreach services.

Although the trial commenced in two areas of PNG, East Sepik Province and Madang Province, in 2008, the East Sepik site was closed due to a lower than excepted malaria incidence. The acceptability research therefore focused upon Madang Province, a mainly coastal lowland (0-500 m) with a high year-round malaria transmission rate. The study was carried out about 60 km north of Madang town.

PNG is renowned for its linguistic and cultural diversity, and in the study area six languages coexist. However, this contrasts with the lack of socio-economic differentiation in rural areas. In Madang province, subsistence farming of a range of crops (including sweet potatoes, greens and tropical fruits) is the main livelihood activity. In addition, both men and women migrate to Mandang town, which is one of the few urban centres in PNG.

## Methods

The methods employed in this study were based on those used in the acceptability studies carried out in sub-Saharan Africa [9-11]. In PNG, emphasis was placed on a questionnaire survey of mothers whose infants participated in the IPTi trial. Additional individual in-depth interviews and focus group discussions with mothers, health workers, village reporters (community members employed by the IPTi trial recording study drug adherence and the occurrence of adverse events), opinion leaders and community members were also undertaken in order to triangulate findings from the questionnaires. The topics explored in the data collection tools were drawn from previous studies and modified to suit the context. These topics included, amongst others: knowledge, understanding and attitudes regarding vaccination; vaccination behaviour; experiences of vaccination; and, knowledge and understanding of IPTi. The behaviour of health workers and participant mothers was also observed during delivery of IPTi and field notes taken.

Respondents were drawn from four villages within the IPTi trial recruitment area. Initial sampling was based on convenience, e.g. mothers who were willing to be interviewed were recruited through study clinics. As the study developed theoretical sampling was used: participants were recruited on the basis of the research team's developing understanding of the field and the questions that emerged from the ongoing analysis of already collected data. The numbers of questionnaires, interviews and FGDs (see Table 1), were determined by the point of saturation (when no more novel information emerged). Prior to their participation, informed consent was obtained from all study respondents in accordance with local ethics committee regulations. Interviews were carried in Tok Pisin (formerly, Pidgin English) or English, and with the agreement of respondents, recorded and transcribed verbatim, and, when necessary, translated into English by field workers for analysis.

**Table 1:** Study respondents and data collection tools.

Type of respondent	**Data collection tool**^**a**^	N
Participant mothers	IDI	23
	QNN	213

Mother refusers	IDI	2
Health workers	IDI	5

Village reporters	IDI	2

Opinion leaders	IDI	2

Community members	FGD	6

^a^IDI: in-depth interview; QNN: questionnaire; FGD: focus group discussion

Qualitative data analysis was carried out employing a Grounded Theory approach [12], with more abstract generalizations emerging from the data based on the coding of emerging themes using QSR Nvivo 2. Descriptive statistics from the qualitative data were generated using SPSS 14.0.

Overall ethics clearance for the acceptability research was obtained from the Ethics Committee of the Comité Ético de Investigación Clínica of the University of Barcelona. Separate local ethical clearance was obtained from the PNG Medical Research Advisory Committee and the PNG Institute of Medical Research Institutional Review Board (IRB), approval number 0807.

## Results

### Acceptability and adherence

A number of key factors have emerged from the data that influence the acceptability of IPTi in positive or negative ways. These factors relate to local attitudes towards infant care and vaccinations, the clinic environment, the IPTi regimen and the nature of the clinical trial context.

#### Local health culture and the routinization of EPI

The interviews with mothers whose infants participated in the study indicate that there is common awareness of and demand for EPI vaccinations. Although the mothers were unable to name all the diseases that vaccination prevents (56% [119/213] could name some), they knew that vaccination prevented diseases or provided protection (91% [194/213]), were positive towards vaccination and often distressed by the possibility that their children may miss out on vaccination. Although over 20% (47/213) of mothers stated that vaccination provides prevention against malaria, it is unclear to what extent this was due to confusion with the IPTi study. Vaccinated children were viewed as healthy; indeed keeping the child healthy was cited as the reason for vaccinating children by over 40% of mothers (87/213). There was debate about whether vaccination provided complete or partial prevention for diseases, with general opinion leaning towards partial prevention: vaccination gave some protection, but complete prevention depends on other factors; the disease would develop more slowly giving mothers chance to go to the clinic; or, the child could be sick again in the future.

Reports of vaccination-related side effects were minimal (2% [5/213] of participant mothers reported side effects), and descriptions focused on mild fever or passing distress. Although relatives and neighbours were said to complain occasionally of children crying after vaccination, mothers were able to dismiss these complains and they did not prevent them from vaccinating their children. Children receiving multiple injections during a single clinic visit was said to cause concern amongst mothers and relatives. Sometimes relatives questioned mothers about the number of injections that the child had received and said that two was too many. One mother reported getting angry after seeing blood drip down her child's arm after vaccination.

In spite of the concerns about multiple injections, very few mothers reported factors preventing them from vaccinating their children (one reported that if she was too busy she may be unable to take her children, another that her husband could prevent her, one mentioned transport problems and one described how she would not be able to go if she was sick). However, in the qualitative interviews, other factors mentioned included: health workers reprimanding mothers for missing previous appointments; difficulties in meeting the costs levied at the clinic; and, laziness.

Although the data suggest that mothers are responsible for the healthcare of the children, taking them to the clinic and caring for them in the home, fathers often play the role of ultimate decision-maker, to whom women have to seek permission to travel to the clinic. Fathers also take a more prominent role in emergencies. Whilst respondents did not report that husbands prevented them from vaccinating their children, there were scattered reports, offered by mothers, of other women's husbands preventing their wives from participating in the IPTi trial. However, one health worker suggested that this might be an easy excuse to avoid refusing directly. The women who participated in the trial reported having made the decision to participate with their husbands, who agreed because they saw the benefits.

The data revealed only scattered accounts of the use of non-biomedical means of preventing disease: two mothers reported using "paw paw" seeds to prevent disease, and one mother specifically mentioned preventing malaria; one mother referred to herbs more generally; and, a health worker described the use of amulets for spiritual protection by members of the local Catholic church. Herbal remedies were more popular for curing rather than preventing: respondents described a range of remedies for mild diseases such as cough, cold, diarrhoea and fever. Mothers, however, preferred that serious diseases be dealt with in the clinic.

#### IPTi drugs, adherence and reasons for refusal

Two-thirds of mothers interviewed knew that the IPTi trial involved malaria prevention (67% [143/213]) and almost three-quarters knew they were participating in research (74% [157/213]). However, the vast majority (87% [186/213]) were unsure about the names of the drugs used, and only four named Fansidar (the brand name for SP) as one of the study drugs. Nevertheless, the mothers were almost universally content with their experiences of the trial: the IPTi drugs, the outreach clinics and the free treatment. Mothers reported a decrease in malaria amongst the participating infants and commented more generally on the health of the infants, comparing it favourably with that of other children who did not participate. They also reported that study participation had brought broader benefits as they travelled less often to the clinic and had more time to do other activities such as work. Indeed, mothers commonly enrolled more that one of their children in the study. Trial staff were considered diligent and caring, compared to other health staff who were rude, slow to deal with mothers, and did not give proper care. One mother conceded that this might be due to the health workers' heavy workload. A common concern expressed by respondents was what would happen when the IPTi trial came to an end in the area.

Reports of side effects from the IPTi drugs were also rare: two mothers referred to the child's body being weak, but that they quickly recovered; a third, described how her child was seen by one of the trial doctors after suffering a reaction to the trial drugs. In none of the cases was the mother discouraged from continuing her participation.

IPTi was delivered in three doses at four intervals to infants who participated. Two doses were left with the mother to give to the child the following days. Fifteen percent of mothers (31/213) reported that they sometimes did not give the IPTi drug as instructed. The majority, who reported having followed the instructions, did so out of the belief that the drugs would help the child and not doing so would be akin to neglect or due to pressure from health workers.

Respondents reported that during the trial and particularly the recruitment phase there were reports that blood sampled from participants was being sold by the local research institute. People with less experience of research, such as men, the elderly and the residents of remote villages, were said to spread these rumours. Indeed, the majority of participant mothers surveyed reported that the blood was used for diagnostic purposes (62% [133/213] referred specifically to testing for malaria). One woman, who refused to participate in the IPTi trial, did so because she believed that the blood would be used for transfusion, something that she disagreed with. Although there were concerns from two participants about their children losing blood and 16% (33/213) of mothers were unsure about the purpose of the blood sampling, many mothers paid little attention to these complains. The majority of the mothers who refused to participate or dropped out from the study did so because of the rumours associated with blood sampling and not because of IPTi per se.

### The influence of IPTi on disease prevention

There is no evidence that IPTi had a negative impact upon EPI adherence. Given the support that the staff employed by the clinical trial gave to the local healthcare system, in the local clinics and at monthly outreach visits to the communities, it is likely that EPI adherence increased during the trial. Furthermore, all participant mothers were positive about their participation.

Participant mothers perceived IPTi as offering complete prevention from malaria: 98% (208/213) of mothers stated that after IPTi their children would not get malaria. This finding is however contradicted by the extent of bed net usage: over three-quarters (77% [163/213]) of participant mothers reported using bed nets as a means of malaria prevention. There was however debate about the effectiveness of bed nets. The new bed nets that were being handed out to trial participants were considered effective at repelling the mosquitoes, in contrast to the less useful older nets. One health worker attributed the popularity of bed nets, to previous campaigns of mosquito eradication that had left a strong message about the importance of preventing mosquito bites.

## Discussion

In a similar fashion to acceptability studies undertaken in Africa [9], the data from PNG indicate a general concern about infant health and that mothers were generally motivated to comply with prevention practices, regardless of whether they fully understood them or not. Although mothers who participated in the IPTi trial were often not entirely clear about the diseases that vaccinations prevented, they viewed EPI non-compliance as neglect. This was based on the general distinction that they drew between the health of un-vaccinated and vaccinated children. Although references to traditional prevention practices were very rare in the PNG data (a finding which differentiates PNG from other sub-Saharan African sites [9-11]) the observed benefits of vaccination, together with the general concern about infant health, formed a favourable context for the acceptance and routinization of both EPI and IPTi.

The presence of the IPTi trial in PNG did not negatively affect participants' attitudes towards EPI vaccinations, nor did it influence their other malaria prevention practices, as was the case in the sub-Saharan African sites [9-11]. However, whilst in the sub-Saharan African sites, the preventive healthcare practices, including IPTi, were almost always considered as bestowing only partial protection, in PNG mothers viewed IPTi as providing complete protection from malaria. Although one may expect a decrease in bed net usage to accompany this finding, no such reduction was reported. This may be the result of the general compliance with preventive practices or to avoid nuisance mosquito bites. However, if IPTi were implemented across PNG and delivered as part of EPI further research on bed net usage would be useful to confirm this finding.

One of the main determinants of acceptability is whether recipients and other community members perceive the intervention as performing as they think it is supposed to, and whether the benefits are perceived to outweigh the disadvantages. In the case of the clinical trial of IPTi in PNG, participant mothers understood that IPTi was intended to prevent malaria in infants and they identified a positive effect upon the health of participating infants and perceived a decrease in malaria incidence. In a similar fashion to the clinical trial sites, where acceptability studies have been undertaken (Kenya, north-eastern Tanzania, Mozambique and Gabon [9-11]) participant mothers and other community members viewed the benefits of trial participation, which included the free high-quality health care provided by the trial staff at local clinics and during outreach services, and the perceived reduction in malaria amongst participating infants as outweighing any concerns about the blood sampling and associated rumours of "blood selling".

Because, in clinical trial settings, it is difficult to isolate the advantages and disadvantages linked to trial from those linked to the intervention, it is challenging to distinguish what precisely motivates mothers to attend clinic and adhere to treatment, and this makes it difficult to assess how they would respond in a more routine implementation setting. However, given the similarities with the response in the intervention sites in Africa it seems likely that the response in "natural" settings will not be very different. In the intervention studies with SP in Malawi and Ghana, 'IPTi was simply an unnoticed addition to an already routinized EPI' [9].

During the PNG trial of IPTi participants had greater opportunity for non-adherence than in the other clinical trials of IPTi: mothers were given the two last doses of the trial drug to administer themselves at home. Although perhaps inflated by desirability bias, the majority of mothers reported having always given the doses as instructed, motivated by the benefits they perceived that the previous doses had proportioned their child (or children). Although a single dose drug regimen for IPTi would be ideal, in order to avoid potential non-adherence, the results from this study suggest that if they perceive benefits, mothers are prepared to administer drugs for prevention unsupervised. Data from other acceptability studies, where health workers sometimes provided IPTi drugs for mothers to administer at home, despite being instructed not to, suggest that some mothers did give the drugs correctly. Other mothers forgot and some, given that they thought the drug was to cool the post-vaccination fever, only gave if the child had fever [9,11]. The latter indicates the importance of providing, in addition to the medication, clear, relevant information.

Respondents' concerns about the care that their infants would receive when the trial ends highlight the broader issue of the feasibility of implementing IPTi in PNG and in other countries with resource-poor health systems. Research in sub-Saharan Africa has, however, shown IPTi to be a cost-effective intervention [13] and pilot implementation studies undertaken by UNICEF in six African countries indicated that IPTi with SP was a feasible intervention, reaching 97% of immunized infants [14]. In spite of these, findings, efforts are required to address the poor coverage and timeliness of childhood immunization outside of the urban centres in PNG [15]. Furthermore, in a third of cases reported in this study, non-vaccination was attributed to problems with the health services [15], which illustrates the need for investment in the provision of MCH outreach services. Although, the data presented provide some insight into health workers' behaviour towards mothers attending MCH for immunization and its effect on immunization adherence, this requires further in-depth study.

The data collected in PNG illustrate additional, striking similarities to those from acceptability studies undertaken in sub-Saharan Africa. For example, mothers in all sites were familiar with vaccination as prevention yet uncertain regarding particular diseases prevented and the nature of the prevention. Also the key barriers to attending clinics, mainly distance and transport and treatment costs, were common in sub-Saharan Africa and PNG. There were also common complaints about (non-trial) health staff that criticize and chastise mothers in the clinic [16]. The data also suggest that the role of fathers in infant care is similar: although fathers play a relatively minor role in day-to-day childcare, when more serious matters arise, such as participation in a clinical trial or severe illness, they take the role of decision-makers. Even the nature of the rumours that circulated around the clinical trial of IPTi in PNG, relating to the selling of the blood sampled, resonated strongly with the rumours encountered in other trial sites [9,10].

### Strengths and limitations of the study

A clinical trial is not the ideal context to investigate the acceptability of any intervention as many of the factors that affect a community's response to the intervention itself are influenced by the presence of the trial. However, in the case of IPTi in PNG, it was the only way of collecting acceptability data early enough to be able to make recommendations for improving access and long-term adherence during roll out. Using a range of methods (including direct observation of behaviour) and interviewing a range of respondents, enabled the triangulation of findings and ensured their reliability.

## Conclusions

The findings presented complement a broader programme of social science research undertaken in sub-Saharan Africa [9]. The inclusion of PNG as an additional site for acceptability research enables comparisons to be made between this very different social and cultural context and the sites in sub-Saharan Africa. The results of the acceptability research in PNG are remarkably similar to those from the African sites and this allows tentative generalization regarding IPTi implementation beyond Africa. Firstly, IPTi seemingly fits well with local health cultures and appears to be accepted easily. Secondly, there is little evidence that IPTi has negative impacts on attitudes to EPI or EPI adherence. Furthermore, although in PNG mothers described IPTi as complete malaria prevention this did not, as in other African sites, influence existing malaria prevention and treatment-seeking behaviour. In practical terms, the degree of similarity across the sites suggests that IPTi would require minimal adjustment in order to be rolled out successfully across diverse social and cultural contexts. The generalizability of findings vindicates the overall research approach of undertaking small-scale social science studies in many sites.

## Competing interests

The authors declare that they have no competing interests.

## Authors' contributions

CP analysed the data and wrote the manuscript. LS trained the fieldworkers. LS, SP, IM, PS and NS planned and supervised data collection. SL and LS carried out data collection. MG and RP designed the IPTi acceptability research programme and the data collection tools. All authors provided comments on the draft manuscript and approved the final version.
